# Supplementary material for: Optical Window to Polarity of Electrolyte Solutions
Source: Molecules. 2023 May 26;28(11):4360. doi: 10.3390/molecules28114360 (PMC10254283; doi:10.3390/molecules28114360)
Supplement: Supplementary file 1 [file molecules-28-04360-s001.zip › molecules-2393510-supplementary.pdf]

Optical Window to Polarity of Electrolyte Solutions (Supplementary Materials)

Omar O'Mari and Valentine I. Vullev*

Materials

Anhydrous solvents ($CHCl_3$, CH_2Cl_2, $(CH_2Cl)_2$, C_6H_5CN, DMF, and CH_3CN) and spectroscopic-grade solvents ($CHCl_3$, CH_2Cl_2, $(CH_2Cl)_2$, C_6H_5CN, DMF, and CH_3CN) were purchased from Fisher Scientific. Two-step synthesis afforded *N*-Phenyl-4-dimethylamino-1,8-naphthalimide (ANI-Ph) as an orange-yellow crystalline solid (60% yield) as we have previously described (*J. Phys. Chem. A* **2009**, *113*, 1259-1267). ^{1}H NMR (400 MHz, DMSO-d_6), δ/ppm: 8.57 (d, 1H), 8.47 (d, 1H), 8.35 (d, 1H), 7.80 (t, 1H), 7.50 (m, 3H), 7.34 (d, 2H), 7.26 (d, 1H), 3.13 (s, 6H) (Figure S1); HRMS: measured *m/z* for $(M+H)^+$ is 317.1293; calculated exact mass of $C_{20}H_{17}N_2O_2^+$ is 317.1285.

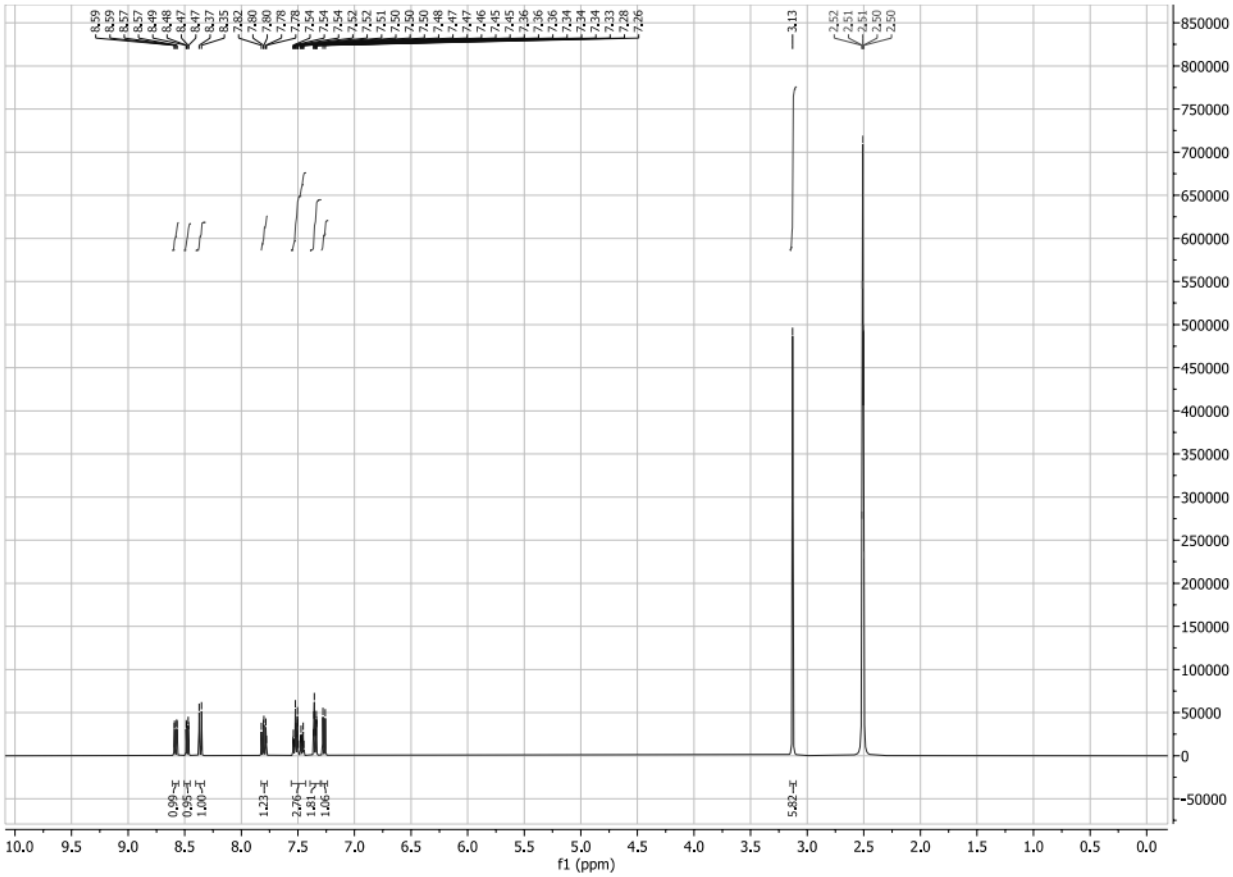

Figure S1. ^{1}H NMR spectrum of ANI-Ph in DMSO-d_6.

Refractometry

The refractive indices of the electrolyte solutions and the solvents (Figure x) are measured at an ambient temperature and pressure using Rudolph Research J357 Automatic Refractometer (Rudolph Research Analytical, Hackettstown, NJ) at the wavelength of the D-line of sodium, 589.3 nm, with an estimated ±0.00002 precision. The refractometer operates on an artificial sapphire prism with automatic temperature control. The liquid samples solutions are allowed to reach thermal equilibrium prior to recording their refractive indices.

Table S1. Experimentally measured indexes of refraction of solutions of $N(n\text{-}C_4H_9)_4PF_6$ with concentrations from 0 to 200 mM.

C_{el} / mM	$CHCl_3$	CH_2Cl_2	$(CH_2Cl)_2$	C_6H_5CN	DMF	CH_3CN
0	1.44524	1.42425	1.34384	1.47899	1.44466	1.43038
25	1.44509	1.42437	1.34454	1.47826	1.44468	1.43036
50	1.44508	1.42438	1.34567	1.47786	1.44463	1.43045
75	1.44502	1.42454	1.34624	1.47770	1.44461	1.43046
100	1.44497	1.42474	1.34706	1.47734	1.44462	1.43050
125	1.44494	1.42491	1.34784	1.47700	1.44456	1.43046
150	1.44488	1.42524	1.34769	1.47660	1.44447	1.43051
175	1.44486	1.42540	1.34842	1.47554	1.44446	1.43052
200	1.44474	1.42554	1.35066	1.47505	1.44439	1.43052

Optical Spectroscopy

Steady-state absorption spectra are recorded in a transmission mode using a JASCO V-670 spectrophotometer (Tokyo, Japan). The steady-state emission spectra are measured, using a FluoroLog-3 spectrofluorometer (Horiba-Jobin-Yvon, Edison, NJ, USA) as previously reported. The optical spectra are recorded in wavelength scales, i.e., absorbance and emission intensity vs. wavelength in nanometers (λ / nm). To convert the thus measured spectra to energy scales, i.e., $\mathcal{E}$, it is important not only to convert the abscissa, i.e., $\mathcal{E} = h\, c\, \lambda^{-1}$, but also to correct (1) the absorbance by dividing it by $\mathcal{E}$, i.e., $A(\mathcal{E}) = A(\lambda)\ \mathcal{E}^{-1}$; and (2) the emission intensity by dividing it by $\mathcal{E}^5$, i.e., $F(\mathcal{E}) = F(\lambda)\ \mathcal{E}^{-5}$. The optical spectra, plotted against $\mathcal{E}$, were fit to sums of Gaussian functions. The positions of the largest-amplitude components were ascribed to the spectral maxima (Figure 5b), which are used for estimating the Stokes' shifts, $\Delta\mathcal{E}$ (Figure 2,3).

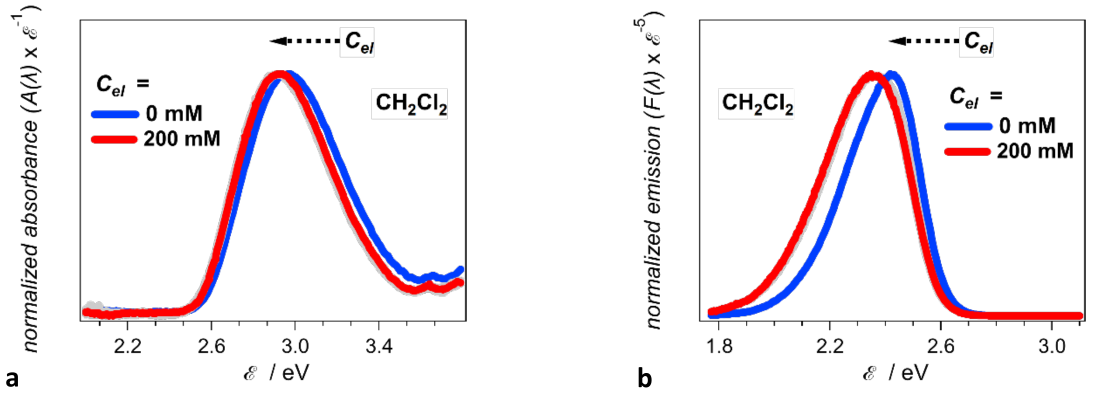

Figure S2. Solvent polarity effect on optical measurements. (a) Normalized absorption spectra of ANI-Ph for CH_2Cl_2 in the presence of different concentrations of N(*n*-$C_4H_9)_4PF_6$. (b) Normalized fluorescence spectra of ANI-Ph for CH_2Cl_2 in the presence of different concentrations of N(*n*-$C_4H_9)_4PF_6$ (λ_{ex} = 421nm).

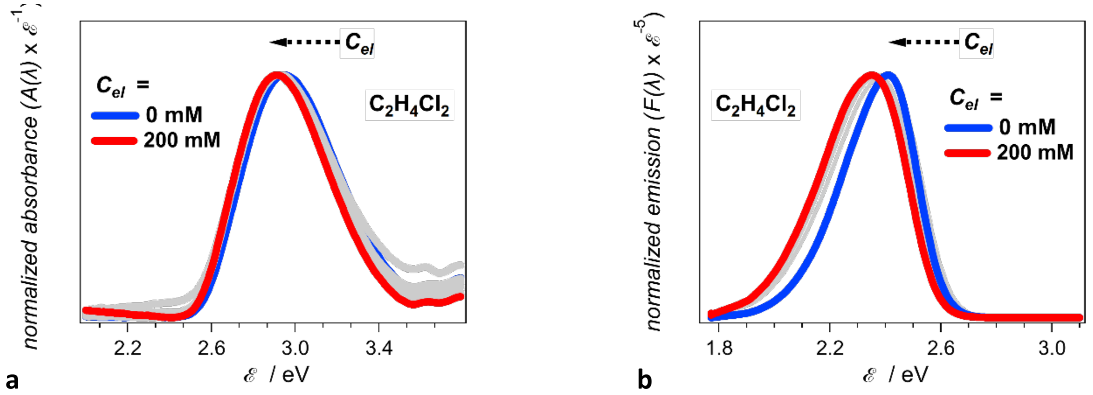

Figure S3. Solvent polarity effect on optical measurements. (a) Normalized absorption spectra of ANI-Ph for $C_2H_4Cl_2$ in the presence of different concentrations of N(*n*-$C_4H_9)_4PF_6$. (b) Normalized fluorescence spectra of ANI-Ph for $C_2H_4Cl_2$ in the presence of different concentrations of N(*n*-$C_4H_9)_4PF_6$ (λ_{ex} = 421nm).

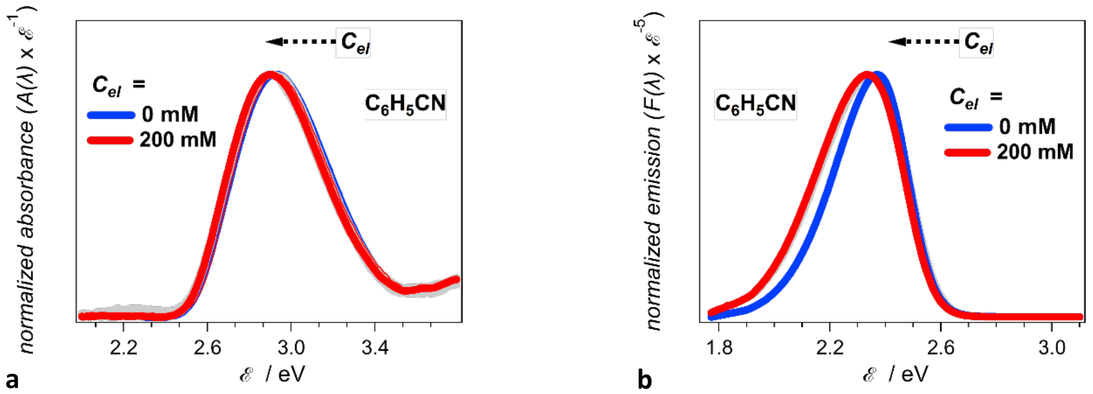

Figure S4. Solvent polarity effect on optical measurements. (a) Normalized absorption spectra of ANI-Ph for C_6H_5CN in the presence of different concentrations of N(*n*-$C_4H_9)_4PF_6$. (b) Normalized fluorescence spectra of ANI-Ph for C_6H_5CN in the presence of different concentrations of N(*n*-$C_4H_9)_4PF_6$ (λ_{ex} = 421nm).

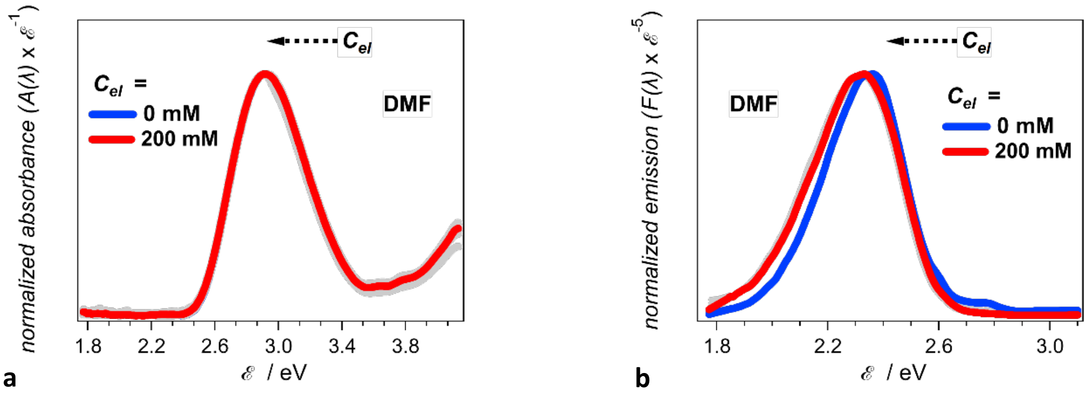

Figure S5. Solvent polarity effect on optical measurements. (a) Normalized absorption spectra of ANI-Ph for DMF in the presence of different concentrations of $N(n\text{-}C_4H_9)_4PF_6$. (b) Normalized fluorescence spectra of ANI-Ph for DMF in the presence of different concentrations of $N(n\text{-}C_4H_9)_4PF_6$ (λ_{ex} = 421nm).

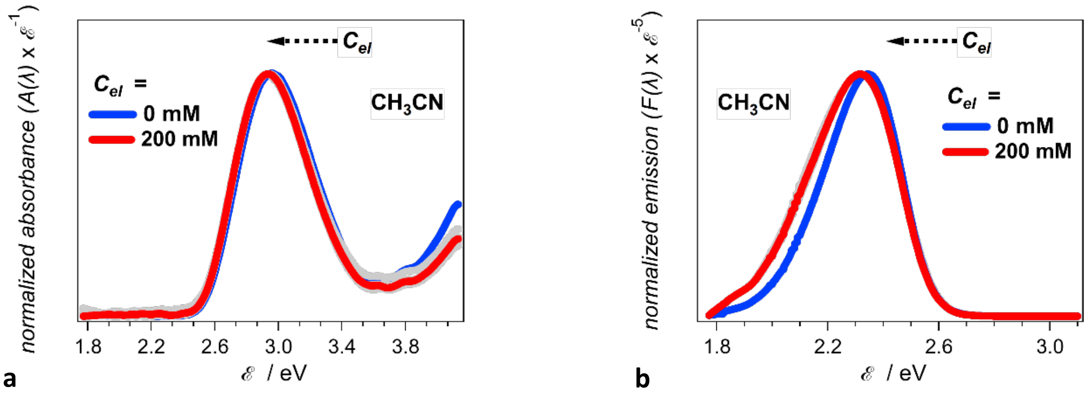

Figure S6. Solvent polarity effect on optical measurements. (a) Normalized absorption spectra of ANI-Ph for CH_3CN in the presence of different concentrations of $N(n\text{-}C_4H_9)_4PF_6$. (b) Normalized fluorescence spectra of ANI-Ph for CH_3CN in the presence of different concentrations of $N(n\text{-}C_4H_9)_4PF_6$ (λ_{ex} = 421nm).

Computational Analysis

The molecular structures of 4-dimethylamino-*N*-phenyl-1,8-naphthalimide (ANI-Ph), are optimized using the Gaussian 09 program package within the Hartree-Fock (HF) theory framework, employing the 6-31G(d,p) basis set. For the excited-states' calculations of ANI-Ph, we resort to the configuration interaction singles (CIS) method using single-excitation CI, i.e., CI-Singles. Solvation effects are implemented with the integral equation formalism variant of the polarizable continuum model (IEFPCM).

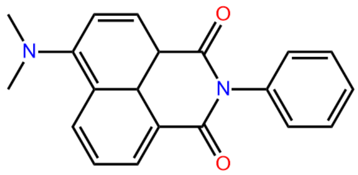

Figure S7. Structure of ANI-Ph.

From the optimized structures, we estimate the permanent electric dipole moments of the ground and the fluorescent excited state. The dipoles of the ground and the excited state are practically parallel, and the solvents have negligible effects on their magnitudes and orientation (Table 1).

The natural transition orbitals (NTOs) represent the electronic transition of optical excitation and emission, with occupation numbers of 0.97 or higher. Varying the solvent has minimal impacts on the NTOs (Figures 1, S8).

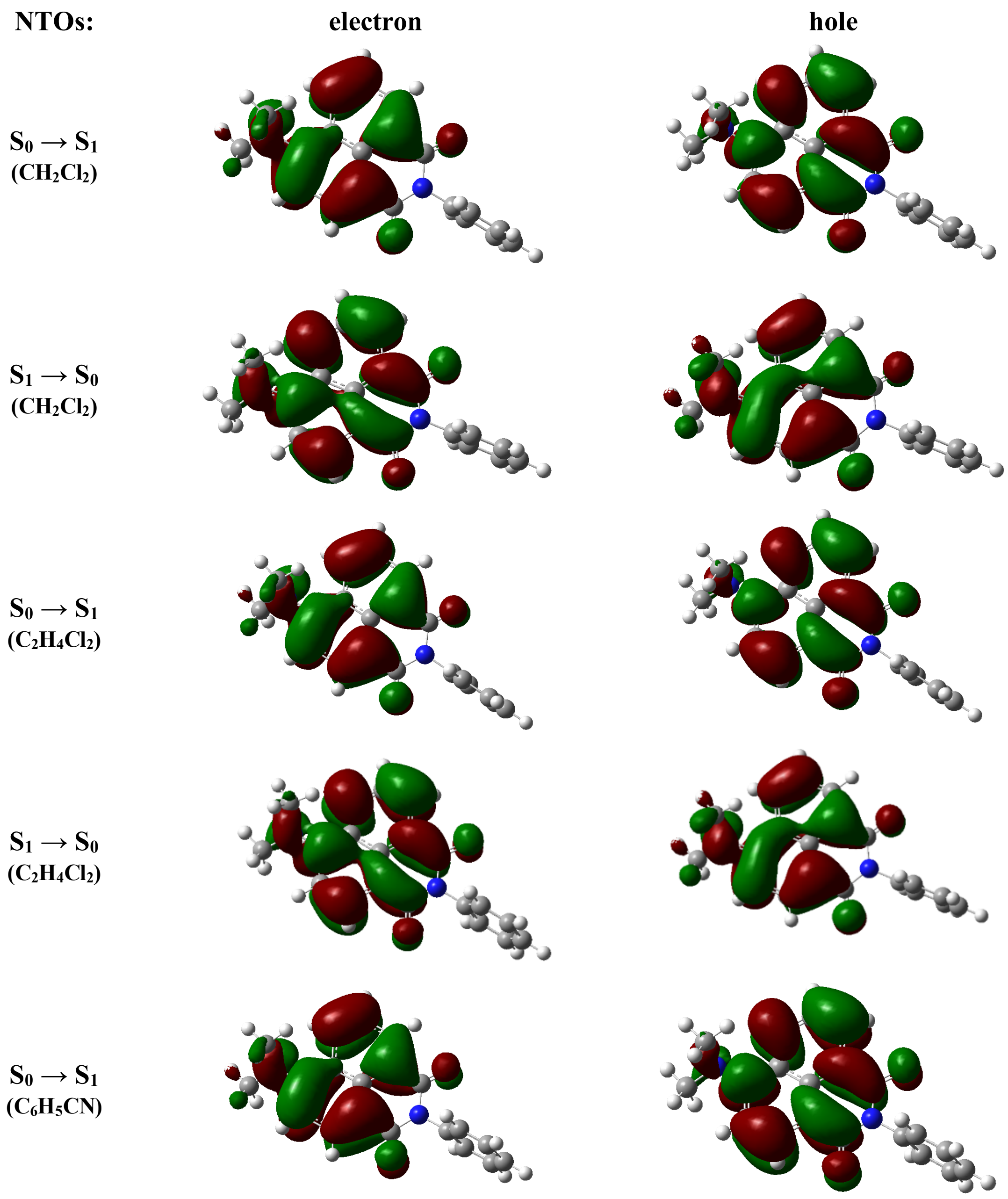

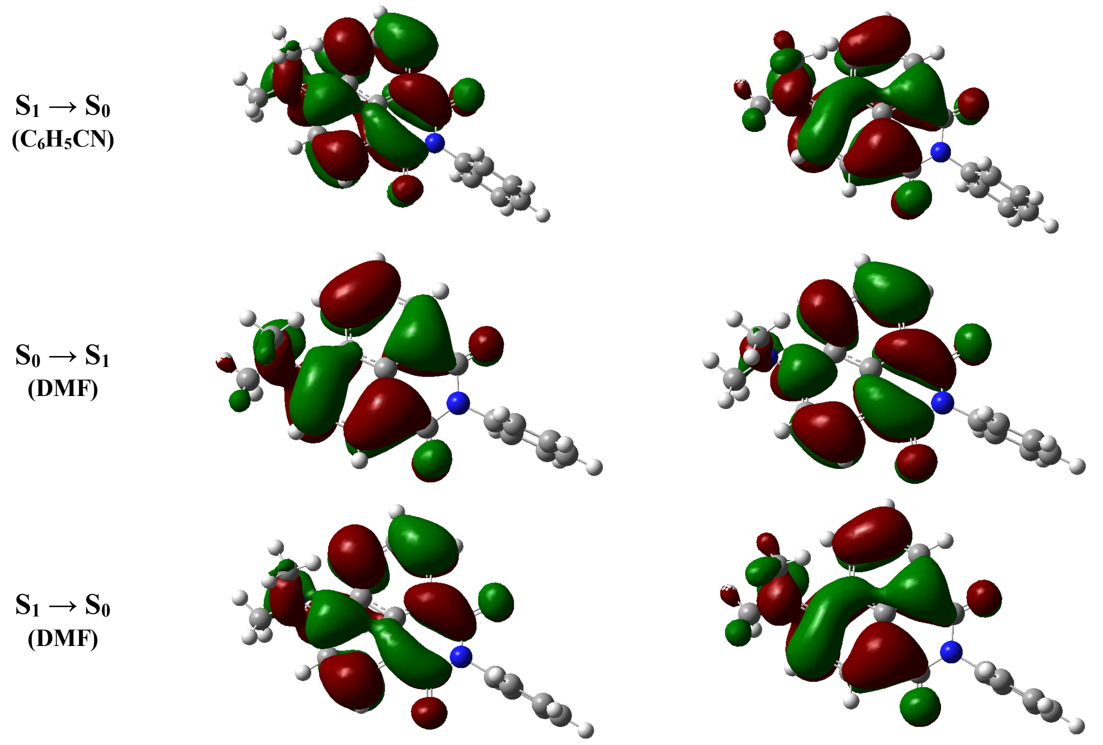

Figure S8. Natural transition orbitals (NTOs) for optical absorption, $S_0 \rightarrow S_1$, and emission, $S_1 \rightarrow S_0$, for CH_2Cl_2, $(CH_2Cl)_2$, C_6H_5CN, and DMF.

Table S2. XYZ atomic coordinates for ANI-Ph in $CHCl_3$, CH_2Cl_2, $(CH_2Cl)_2$, C_6H_5CN, DMF, and CH_3CN as implicit solvents.

Optimized Ground State in $CHCl_3$					Optimized Excited State in $CHCl_3$			
C	-1.0357	0.349595	-0.06668		C	-1.01941	0.345093	-0.07208
C	-2.43134	0.521458	-0.09785		C	-2.44443	0.519023	-0.11492
C	-2.94228	1.839981	-0.19596		C	-2.93973	1.803393	-0.27073
C	-2.10976	2.919882	-0.21406		C	-2.07633	2.926361	-0.28023
C	-0.71636	2.740636	-0.14215		C	-0.72522	2.77477	-0.17108
C	-0.18881	1.48165	-0.07755		C	-0.17928	1.474467	-0.08107
C	-0.47906	-0.95059	-0.05126		C	-0.47014	-0.9401	-0.08501
C	-1.30516	-2.0359	-0.07621		C	-1.30266	-2.09468	-0.1681
C	-2.70308	-1.88117	-0.08305		C	-2.65155	-1.95274	-0.16846
C	-3.27811	-0.63791	-0.07249		C	-3.27414	-0.66686	-0.06371
C	1.281899	1.312084	-0.02991		C	1.269298	1.317082	-0.02655
N	1.770473	0.009328	-0.00734		N	1.763912	0.013739	-0.0071
C	0.984749	-1.1411	-0.02428		C	0.982593	-1.12887	-0.04946
C	3.200663	-0.16273	0.029027		C	3.192419	-0.15768	0.043975
C	3.909409	-0.25163	-1.15481		C	3.915107	-0.24273	-1.13198
C	5.284122	-0.41789	-1.11842		C	5.289177	-0.41008	-1.08116
C	5.942893	-0.49468	0.098573		C	5.934908	-0.49202	0.142567
C	5.224987	-0.40442	1.280593		C	5.203705	-0.4057	1.316709
C	3.850128	-0.23805	1.247194		C	3.829355	-0.23837	1.268667
O	1.495695	-2.2254	-0.01512		O	1.482885	-2.22758	-0.05151
O	2.034578	2.244248	-0.01289		O	2.030195	2.254921	0.000716

N	-4.67498	-0.44078	-0.05324		N	-4.62406	-0.57303	0.067157
C	-5.20672	-0.05453	1.250341		C	-5.24592	0.271238	1.084009
C	-5.48466	-1.4708	-0.67904		C	-5.48868	-1.64724	-0.38526
H	-4.00277	1.978016	-0.27591		H	-3.99058	1.964162	-0.40521
H	-2.51346	3.912325	-0.29341		H	-2.50601	3.907535	-0.37074
H	-0.0572	3.587566	-0.15214		H	-0.0632	3.617786	-0.16861
H	-0.87882	-3.0211	-0.0721		H	-0.84421	-3.06202	-0.20548
H	-3.31554	-2.76166	-0.07688		H	-3.27016	-2.82811	-0.18195
H	3.389555	-0.19163	-2.09386		H	3.405732	-0.17809	-2.07641
H	5.837394	-0.48745	-2.03777		H	5.852479	-0.47618	-1.99477
H	7.010209	-0.624	0.125641		H	7.001863	-0.62198	0.180876
H	5.732162	-0.46332	2.226881		H	5.700285	-0.46828	2.268483
H	3.284606	-0.16725	2.158692		H	3.253822	-0.17027	2.174075
H	-4.61009	0.731728	1.690229		H	-4.5156	0.901721	1.561395
H	-5.2277	-0.89517	1.944732		H	-5.69898	-0.36335	1.841954
H	-6.21806	0.314058	1.126236		H	-6.02244	0.888635	0.646507
H	-5.0792	-1.72264	-1.65023		H	-5.1247	-2.062	-1.31409
H	-6.4862	-1.08169	-0.81934		H	-6.47573	-1.23718	-0.56026
H	-5.56241	-2.38167	-0.0848		H	-5.58063	-2.44222	0.353314
**Optimized Ground State in CH_2Cl_2**					**Optimized Excited State in CH_2Cl_2**			
C	-1.03524	0.349196	-0.06726		C	-1.01903	0.34456	-0.07269
C	-2.43092	0.521046	-0.09797		C	-2.44398	0.519059	-0.11399
C	-2.94177	1.839588	-0.19639		C	-2.93921	1.802724	-0.27038
C	-2.10953	2.919911	-0.21485		C	-2.07552	2.925916	-0.28042
C	-0.71638	2.740672	-0.14303		C	-0.72464	2.774654	-0.17142
C	-0.18853	1.481463	-0.07835		C	-0.17821	1.474576	-0.08166
C	-0.47863	-0.95105	-0.05243		C	-0.46978	-0.94061	-0.08757
C	-1.30537	-2.03638	-0.07837		C	-1.30299	-2.09502	-0.17302
C	-2.70292	-1.88202	-0.08525		C	-2.65152	-1.95322	-0.17162
C	-3.27822	-0.63844	-0.07286		C	-3.27506	-0.66784	-0.06236
C	1.281303	1.31141	-0.0312		C	1.268977	1.316502	-0.02799
N	1.76986	0.009192	-0.00785		N	1.763302	0.013839	-0.00816
C	0.984213	-1.14082	-0.02495		C	0.982031	-1.12839	-0.05149
C	3.199933	-0.16278	0.029365		C	3.191634	-0.15776	0.04433
C	3.909424	-0.25143	-1.15432		C	3.91576	-0.24182	-1.1311
C	5.284388	-0.41748	-1.11695		C	5.29004	-0.40905	-1.0787
C	5.94248	-0.49416	0.100668		C	5.934448	-0.49172	0.145928
C	5.223695	-0.40414	1.28243		C	5.201728	-0.40645	1.319453
C	3.848574	-0.23799	1.248259		C	3.827147	-0.23929	1.269994
O	1.497081	-2.22526	-0.01541		O	1.484761	-2.22732	-0.05387
O	2.03569	2.243367	-0.01509		O	2.032232	2.254445	-0.00149
N	-4.67431	-0.44173	-0.05376		N	-4.62263	-0.57629	0.072512
C	-5.2065	-0.0455	1.247272		C	-5.24799	0.280059	1.077616
C	-5.48482	-1.47701	-0.67069		C	-5.48874	-1.65179	-0.37632
H	-4.00215	1.978375	-0.27587		H	-3.98987	1.964693	-0.40335
H	-2.51343	3.912168	-0.29416		H	-2.50552	3.906905	-0.37076
H	-0.05824	3.588362	-0.15327		H	-0.06407	3.618828	-0.16918
H	-0.88032	-3.02212	-0.07477		H	-0.84629	-3.06299	-0.21368

H	-3.31487	-2.7627	-0.0798		H	-3.26959	-2.82887	-0.18623
H	3.390186	-0.1914	-2.09374		H	3.407584	-0.17666	-2.07619
H	5.838283	-0.48686	-2.0359		H	5.854424	-0.47449	-1.99164
H	7.009754	-0.62326	0.128415		H	7.001313	-0.62155	0.185393
H	5.73027	-0.463	2.229004		H	5.69717	-0.46974	2.271728
H	3.28245	-0.16731	2.159423		H	3.250598	-0.1721	2.174871
C	-1.03524	0.349196	-0.06726		H	-4.51687	0.904231	1.561864
C	-2.43092	0.521046	-0.09797		H	-5.71627	-0.34784	1.831375
C	-2.94177	1.839588	-0.19639		H	-6.01283	0.903377	0.628423
C	-2.10953	2.919911	-0.21485		H	-5.12686	-2.06827	-1.30508
C	-0.71638	2.740672	-0.14303		H	-6.47579	-1.24142	-0.54929
C	-0.18853	1.481463	-0.07835		H	-5.57828	-2.44443	0.364526
**Optimized Ground State in $(CH_2Cl)_2$**					**Optimized Excited State in $(CH_2Cl)_2$**			
C	-1.03517	0.349149	-0.06735		C	-1.01897	0.344483	-0.07273
C	-2.43085	0.521012	-0.09795		C	-2.44391	0.519076	-0.11382
C	-2.94169	1.839567	-0.19637		C	-2.93912	1.802655	-0.27025
C	-2.10947	2.919939	-0.21489		C	-2.07539	2.925867	-0.28031
C	-0.71635	2.740686	-0.14315		C	-0.72453	2.774636	-0.17135
C	-0.18847	1.481441	-0.07849		C	-0.17805	1.474583	-0.08169
C	-0.47858	-0.95111	-0.05264		C	-0.46974	-0.94069	-0.08788
C	-1.30542	-2.03643	-0.07873		C	-1.30306	-2.09506	-0.17365
C	-2.70291	-1.88211	-0.0856		C	-2.65155	-1.95327	-0.17206
C	-3.27823	-0.63848	-0.0729		C	-3.2752	-0.66796	-0.0622
C	1.281232	1.311297	-0.03147		C	1.268942	1.316396	-0.02816
N	1.769767	0.009151	-0.00796		N	1.763217	0.013828	-0.00829
C	0.984122	-1.14079	-0.02508		C	0.981941	-1.12834	-0.05174
C	3.199816	-0.16282	0.02941		C	3.191522	-0.1578	0.044362
C	3.909453	-0.25144	-1.15423		C	3.915855	-0.2415	-1.13101
C	5.284455	-0.41744	-1.11668		C	5.29017	-0.4087	-1.07841
C	5.942413	-0.49408	0.101053		C	5.934396	-0.49165	0.146324
C	5.223466	-0.40409	1.282757		C	5.201461	-0.40675	1.319779
C	3.848305	-0.23799	1.248431		C	3.826842	-0.23963	1.270152
O	1.49725	-2.22526	-0.0155		O	1.48501	-2.22731	-0.05417
O	2.03588	2.243214	-0.01555		O	2.032541	2.254353	-0.00179
N	-4.67423	-0.44181	-0.0538		N	-4.62245	-0.5767	0.07316
C	-5.20645	-0.0444	1.246953		C	-5.24832	0.281222	1.076687
C	-5.48485	-1.47777	-0.6696		C	-5.48875	-1.65236	-0.37525
H	-4.00205	1.97848	-0.27573		H	-3.98975	1.964805	-0.403
H	-2.51339	3.912175	-0.29416		H	-2.50543	3.906836	-0.37058
H	-0.05835	3.588479	-0.15343		H	-0.06417	3.618975	-0.1691
H	-0.88056	-3.02226	-0.07523		H	-0.84662	-3.06313	-0.21475
H	-3.31481	-2.76281	-0.08028		H	-3.26954	-2.82895	-0.18683
H	3.390328	-0.19141	-2.09372		H	3.407843	-0.17613	-2.07618
H	5.838465	-0.48679	-2.03555		H	5.854708	-0.47387	-1.99128
H	7.009683	-0.62312	0.12893		H	7.001253	-0.62144	0.185929
H	5.729927	-0.46292	2.229388		H	5.696747	-0.47028	2.272112
H	3.282064	-0.16732	2.159529		H	3.250148	-0.17272	2.174965
H	-4.60819	0.742763	1.682654		H	-4.51716	0.904635	1.561834

H	-5.23076	-0.88076	1.946109		H	-5.71855	-0.34582	1.829881
H	-6.21637	0.326289	1.118834		H	-6.01165	0.905224	0.625929
H	-5.08179	-1.7372	-1.6398		H	-5.12711	-2.06903	-1.30401
H	-6.48685	-1.09014	-0.81007		H	-6.47578	-1.24193	-0.54796
H	-5.56015	-2.38346	-0.06762		H	-5.57797	-2.44471	0.365869
**Optimized Ground State in C_6H_5CN**					**Optimized Excited State in C_6H_5CN**			
C	-1.03483	0.34891	-0.06786		C	-1.01869	0.344089	-0.07283
C	-2.43053	0.520887	-0.09779		C	-2.44357	0.519176	-0.11289
C	-2.94124	1.839526	-0.19598		C	-2.93866	1.802383	-0.26939
C	-2.10911	2.920101	-0.21489		C	-2.07469	2.925651	-0.27943
C	-0.71612	2.740725	-0.14378		C	-0.72393	2.774534	-0.17065
C	-0.18816	1.481279	-0.07937		C	-0.17723	1.474585	-0.08159
C	-0.47838	-0.95143	-0.05371		C	-0.46956	-0.94111	-0.08947
C	-1.30572	-2.03672	-0.08043		C	-1.30342	-2.09526	-0.17673
C	-2.70296	-1.8825	-0.08719		C	-2.65172	-1.95343	-0.17402
C	-3.27834	-0.63861	-0.07299		C	-3.27589	-0.66844	-0.06137
C	1.280934	1.310662	-0.03317		C	1.268807	1.315812	-0.0288
N	1.769323	0.008844	-0.0087		N	1.762821	0.013687	-0.00905
C	0.983635	-1.14071	-0.02581		C	0.9815	-1.12815	-0.05325
C	3.199243	-0.16317	0.029604		C	3.190991	-0.15813	0.044431
C	3.909724	-0.25194	-1.15374		C	3.916527	-0.23899	-1.13063
C	5.284919	-0.41759	-1.1151		C	5.291025	-0.4059	-1.07704
C	5.942129	-0.49349	0.103262		C	5.934192	-0.49118	0.148282
C	5.222276	-0.40332	1.284589		C	5.200028	-0.40914	1.321357
C	3.846905	-0.23761	1.249332		C	3.825207	-0.24235	1.270852
O	1.497927	-2.22534	-0.01593		O	1.486165	-2.22732	-0.05622
O	2.036837	2.242384	-0.01854		O	2.034053	2.25384	-0.00281
N	-4.67384	-0.44207	-0.05389		N	-4.62167	-0.57849	0.076078
C	-5.20617	-0.03934	1.245597		C	-5.25008	0.286638	1.07216
C	-5.48501	-1.48106	-0.66455		C	-5.48874	-1.65491	-0.37057
H	-4.00153	1.979103	-0.27454		H	-3.98915	1.96542	-0.40114
H	-2.51312	3.912255	-0.29377		H	-2.5049	3.906539	-0.36929
H	-0.05876	3.588987	-0.15436		H	-0.06455	3.619662	-0.16824
H	-0.88183	-3.02295	-0.07744		H	-0.84828	-3.06381	-0.21995
H	-3.31463	-2.76327	-0.08243		H	-3.26944	-2.82925	-0.1895
H	3.391227	-0.19226	-2.09362		H	3.409431	-0.17178	-2.0762
H	5.839591	-0.48709	-2.03354		H	5.856456	-0.46896	-1.98946
H	7.009391	-0.62214	0.131916		H	7.00101	-0.62068	0.188616
H	5.728103	-0.46161	2.231565		H	5.694426	-0.47461	2.273985
H	3.279998	-0.16666	2.160021		H	3.247663	-0.17762	2.175315
H	-4.60751	0.748655	1.679025		H	-4.51896	0.906679	1.561571
H	-5.23142	-0.87361	1.947066		H	-5.72935	-0.33643	1.822622
H	-6.21564	0.331692	1.115686		H	-6.00639	0.913603	0.614011
H	-5.08318	-1.74447	-1.6342		H	-5.1281	-2.0724	-1.29928
H	-6.4872	-1.09408	-0.80511		H	-6.47569	-1.2442	-0.54216
H	-5.55911	-2.38402	-0.0586		H	-5.5766	-2.44594	0.371775
**Optimized Ground State in DMF**					**Optimized Excited State in DMF**			

C	-1.03476	0.348856	-0.06799		C	-1.01863	0.343996	-0.07276
C	-2.43046	0.520866	-0.09773		C	-2.4435	0.519199	-0.11264
C	-2.94114	1.839528	-0.19583		C	-2.93854	1.802331	-0.26919
C	-2.10901	2.92014	-0.21483		C	-2.07452	2.925603	-0.27917
C	-0.71606	2.740728	-0.14393		C	-0.72379	2.774505	-0.17036
C	-0.18808	1.481233	-0.0796		C	-0.17704	1.474574	-0.08144
C	-0.47835	-0.9515	-0.05396		C	-0.46953	-0.94122	-0.08974
C	-1.3058	-2.03678	-0.08082		C	-1.3035	-2.0953	-0.17732
C	-2.70298	-1.88258	-0.08754		C	-2.65177	-1.95346	-0.17439
C	-3.27836	-0.63863	-0.073		C	-3.27604	-0.66853	-0.0612
C	1.280876	1.310504	-0.03365		C	1.268784	1.315665	-0.02879
N	1.769228	0.008755	-0.0089		N	1.762739	0.013634	-0.00918
C	0.983518	-1.1407	-0.02598		C	0.981402	-1.12813	-0.05357
C	3.199116	-0.16327	0.029641		C	3.19088	-0.15823	0.044425
C	3.909812	-0.25216	-1.15361		C	3.916672	-0.23815	-1.1306
C	5.285052	-0.4177	-1.1147		C	5.291216	-0.40496	-1.07684
C	5.942077	-0.49332	0.103824		C	5.934167	-0.49104	0.148576
C	5.221998	-0.40304	1.285046		C	5.199744	-0.40995	1.321596
C	3.846576	-0.23745	1.249545		C	3.824872	-0.24326	1.270956
O	1.498048	-2.22538	-0.016		O	1.486411	-2.22734	-0.05675
O	2.037055	2.242185	-0.01939		O	2.034386	2.253711	-0.00283
N	-4.67376	-0.4421	-0.05388		N	-4.62152	-0.57886	0.07661
C	-5.20609	-0.03831	1.245361		C	-5.25054	0.287827	1.071026
C	-5.48507	-1.48168	-0.6635		C	-5.48873	-1.65545	-0.3697
H	-4.00142	1.979258	-0.27415		H	-3.98899	1.965564	-0.40078
H	-2.51305	3.912281	-0.29359		H	-2.50476	3.906475	-0.36896
H	-0.05883	3.589087	-0.15459		H	-0.06461	3.619804	-0.16787
H	-0.88213	-3.02311	-0.07793		H	-0.84866	-3.06397	-0.22098
H	-3.31461	-2.76336	-0.08289		H	-3.26943	-2.8293	-0.19004
H	3.39147	-0.19263	-2.09359		H	3.409761	-0.1703	-2.07624
H	5.83989	-0.4873	-2.03302		H	5.856836	-0.46732	-1.98919
H	7.009337	-0.62186	0.13268		H	7.000981	-0.62046	0.189026
H	5.727667	-0.46112	2.232114		H	5.69396	-0.47607	2.274266
H	3.279503	-0.16636	2.160126		H	3.247149	-0.17924	2.175362
H	-4.60727	0.749772	1.678383		H	-4.51949	0.907177	1.561391
H	-5.23162	-0.87219	1.94725		H	-5.73179	-0.33438	1.820868
H	-6.21543	0.332902	1.115096		H	-6.00533	0.915384	0.611222
H	-5.0835	-1.74591	-1.63305		H	-5.12823	-2.07314	-1.29836
H	-6.48728	-1.0948	-0.80408		H	-6.47564	-1.24467	-0.54114
H	-5.55893	-2.38409	-0.05675		H	-5.57636	-2.44617	0.372928
**Optimized Ground State in CH_3CN**					**Optimized Excited State in CH_3CN**			
C	-1.03477	0.348861	-0.06798		C	-1.01864	0.344005	-0.07277
C	-2.43047	0.520868	-0.09774		C	-2.44351	0.519196	-0.11266
C	-2.94115	1.839527	-0.19584		C	-2.93855	1.802336	-0.26921
C	-2.10902	2.920137	-0.21484		C	-2.07454	2.925608	-0.2792
C	-0.71607	2.740728	-0.14391		C	-0.7238	2.774509	-0.17039
C	-0.18809	1.481237	-0.07958		C	-0.17706	1.474576	-0.08145
C	-0.47835	-0.9515	-0.05394		C	-0.46953	-0.94121	-0.08971

C	-1.3058	-2.03678	-0.08078		C	-1.30349	-2.0953	-0.17726
C	-2.70298	-1.88257	-0.0875		C	-2.65177	-1.95346	-0.17435
C	-3.27836	-0.63863	-0.073		C	-3.27603	-0.66852	-0.06122
C	1.280882	1.310519	-0.0336		C	1.268786	1.31568	-0.0288
N	1.769237	0.008764	-0.00888		N	1.762747	0.013641	-0.00917
C	0.98353	-1.1407	-0.02596		C	0.981412	-1.12813	-0.05354
C	3.199128	-0.16326	0.029637		C	3.190891	-0.15821	0.044424
C	3.909803	-0.25213	-1.15362		C	3.916659	-0.23824	-1.13061
C	5.285039	-0.41769	-1.11474		C	5.291198	-0.40507	-1.07686
C	5.942082	-0.49334	0.103769		C	5.934169	-0.49105	0.148552
C	5.222024	-0.40306	1.285001		C	5.19977	-0.40986	1.321576
C	3.846608	-0.23746	1.249524		C	3.824904	-0.24316	1.270946
O	1.498037	-2.22538	-0.01599		O	1.486388	-2.22734	-0.0567
O	2.037034	2.242204	-0.01931		O	2.034354	2.253725	-0.00284
N	-4.67377	-0.4421	-0.05388		N	-4.62153	-0.57882	0.076557
C	-5.2061	-0.03841	1.245383		C	-5.2505	0.287713	1.07113
C	-5.48506	-1.48162	-0.6636		C	-5.48873	-1.6554	-0.36978
H	-4.00143	1.979243	-0.27419		H	-3.989	1.96555	-0.40081
H	-2.51306	3.912278	-0.29361		H	-2.50477	3.906481	-0.36899
H	-0.05883	3.589078	-0.15457		H	-0.06461	3.619791	-0.1679
H	-0.8821	-3.02309	-0.07789		H	-0.84862	-3.06395	-0.22087
H	-3.31461	-2.76335	-0.08285		H	-3.26943	-2.8293	-0.18998
H	3.391446	-0.19259	-2.0936		H	3.409731	-0.17046	-2.07623
H	5.83986	-0.48728	-2.03307		H	5.8568	-0.4675	-1.98921
H	7.009342	-0.62188	0.132605		H	7.000983	-0.62048	0.188992
H	5.727709	-0.46117	2.23206		H	5.694004	-0.47591	2.274242
H	3.279551	-0.16639	2.160116		H	3.247197	-0.17907	2.175357
H	-4.6073	0.749669	1.678442		H	-4.51944	0.90713	1.561405
H	-5.2316	-0.87232	1.947233		H	-5.73156	-0.33458	1.821032
H	-6.21545	0.332784	1.115152		H	-6.00543	0.915213	0.611483
H	-5.08347	-1.74577	-1.63316		H	-5.12821	-2.07307	-1.29844
H	-6.48728	-1.09474	-0.80417		H	-6.47565	-1.24463	-0.54124
H	-5.55895	-2.38408	-0.05693		H	-5.57638	-2.44614	0.37282
